# Editorial: Functional near-infrared diffuse optical spectroscopy (fNIRS) to explore mental health - volume II

**DOI:** 10.3389/fpsyt.2024.1467499

**Published:** 2024-08-09

**Authors:** Yu Shang, Ting Li, Chong Huang

**Affiliations:** ^1^ School of Life and Health Technology, Dongguan University of Technology, Dongguan, China; ^2^ Institute of Biomedical Engineering, Chinese Academy of Medical Science and Peking Union Medical College, Tianjin, China; ^3^ College of Engineering, University of Kentucky, Lexington, KY, United States

**Keywords:** mental disorders, fNIRS, cerebral oxygenation, brain connectivity, resting-state brain networks, lateralization difference, cerebral small vessel disease, diffuse correlation spectroscopy

Mental health disorders have been attracting the attention of both scientific and clinical communities, for their increasing rate in recent years and for their growing public burden . On the other hand, the clinical evaluation of mental health is predominantly subjected to questionnaires and score sheets, due to the lack of quantitative approaches. Over the years, functional near-infrared diffuse optical spectroscopy (fNIRS) has been of interest to scientists and clinicians in both brain function and clinical psychiatric fields. Owing to several advantages such as noninvasiveness, low cost and high sensitivity, fNIRS is being adopted more often than before to assess cerebral hemodynamics (e.g., blood oxygenation, blood flow, oxygen metabolic rate), which are highly associated with brain cortex function and symptoms of psychiatric diseases.

As a continuation of the Volume I Research Topic which contains twelve articles published from January 2021 to June 2022, this Volume II Research Topic includes the review and research articles published from November 2022 to May 2024, reporting the fNIRS methods for evaluating mental health and brain function and for investigating psychiatric diseases. The methodologies and protocols used, along with the key discoveries are summarized and highlighted in the following paragraphs.

## Reviews on the use of fNIRS in neurological evaluation

In the article authored by Li et al., the literature on fNIRS from 1990 to 2023 was searched and the top 100 most cited articles were identified. Using the bibliometrics package in R studio and VOSviewer for data analysis and plotting, the characteristics and citation status of these 100 most cited articles were obtained. The results revealed that fNIRS has emerged as a powerful tool in the evaluation of brain cortex functions, with a gradual increase in articles and citations over the years. The future directions for fNIRS research would be the longitudinal assessment of therapeutic effects, such as electrical stimulation, TMS, and the extension from spectroscopy to tomography, i.e., diffuse optical tomography (DOT).

Another review article, authored by Su et al., focused on a literature review on the tracking of neurodevelopmental trajectories in infants and children with and without developmental disorders. The results indicated a general trend of age-related increases in network integration and segregation, interhemispheric connectivity, leftward asymmetry, and differential activation when processing visual, auditory, and tactile information. However, these developmental trajectories are different for children with developmental disorders. These discoveries support the use of fNIRS to track neurodevelopmental trajectories in children, to use fNIRS-related neurobiomarkers for early identification of developmental disorders, and to assess the effects of interventions.

## Studies on the use of fNIRS to evaluate brain cortex function in infants

fNIRS is an infant-friendly neuroimaging tool that enables the monitoring of cerebral hemodynamic responses in the neonatal period. With the advantages of noninvasiveness, portability, low cost and sufficient penetration depth, fNIRS is a promising tool for investigating cortical activations in infants. In a study conducted by Wang et al., gender differences from the resting-state network of infant sleep state were examined by analyzing the open access fNIRS dataset containing task-free hemodynamic activity in 4-month-old infants during sleep. The results showed that female and male infants significantly differed in Power Spectral Density for oxyhemoglobin and deoxyhemoglobin at rest, with stronger differences in the frontoparietal network, somatomotor network, visual network and dorsal network. These differences are attributed to sex differences in the timing or extent of the development of brain networks, indicating the potential to guide the early education of male and female infants.

Furthermore, fNIRS was also utilized by Gao et al. to explore the influence of a bilingual environment on brain development in infants. The functional connectivity of the frontal lobes in 4-month-old infants from different language groups was calculated by wavelet transform coherence, graph theory, and Granger causality processing on fNIRS signals. The results showed that the functional connectivity strength in the left hemisphere was significantly higher than in the right hemisphere. There are significant differences between monolingual and bilingual infant groups in several fNIRS characteristics, suggesting that the left hemispheric lateralization that appeared in a bilingual group would be a useful biomarker to investigate the resting-state brain networks of infants.

## Studies on the use of fNIRS to evaluate brain cortex function in adults

In addition to infants, lateralization differences in functional activity have also been investigated in adults. In a study, conducted by Chen et al., the fNIRS and EEG were simultaneously recorded in healthy adults during Stroop tasks, and the brain connectivity of bilateral frontal lobes was analyzed using Pearson correlation coefficient matrix and wavelet transform coherence approaches. The results from the EEG signals did not present any significant lateralization, while the fNIRS analysis showed that the left frontal lobe plays a leading role in dealing with conflict tasks. These observations in brain connectivity during cognitive conflict processing demonstrated that fNIRS is a valuable tool for identifying the lateralization difference during cognitive or physiological studies.

Smartphones have become the most widely used devices worldwide, but their potential negative effects on physical and mental health have been rarely studied. Liu et al. conducted a study to investigate the impairment of decision-making ability in college students using fNIRS technology. Participants were instructed to perform the Iowa Gambling Task (IGT) on smartphones while fNIRS recorded their prefrontal cortex activity to evaluate their decision-making under risky and uncertain conditions. The outcomes indicated that individuals prone to smartphone addiction tend to make riskier choices in risky situations. In particular, the left dlPFC (dorsolateral prefrontal cortex) exhibited significantly higher activation in the addiction group compared with the control group. These findings support the notion that smartphone addiction may influence decision-making, behaviorally and neurologically, particularly in uncertain contexts, and reinforce the necessity to classify smartphone addiction as a public health issue.

In a study conducted by Powers et al., the prefrontal cortex (PFC) response to infant cues during pregnancy was investigated using fNIRS technology. The hemodynamic responses to an infant cry task, and an infant face task were compared in women who used or did not use cannabis. The authors found preliminary group differences in the dorsomedial PFC when responding to both infant cries and infant sad faces, suggesting that cannabis use during pregnancy may be associated with brain activation in the regions involved in emotional regulation and information processing.

## Studies on the use of fNIRS in the diagnosis of psychiatric diseases

Precise detection of mental impairment and the diagnosis of psychiatric diseases are the main goals of fNIRS for clinical use. Various physiological paradigms, such as verbal fluency tasks (VFT), high-level cognition tasks (HCT), and voluntary breath holding (VBH), have been the most popular protocols to induce brain cortex activation, which is also reflected in this Research Topic. In a study conducted by Wen et al., the protocols of VFT, HCT and VBH were sequentially performed on two patients with cerebral small vessel disease (CSVD) and a healthy subject. Meanwhile, both fNIRS and an emerging technology of diffuse correlation spectroscopy (DCS) were utilized to longitudinally monitor the cerebral oxygenation activation and cerebral blood flow (CBF) responses. The results showed that both CSVD patients exhibited abnormal cerebral oxygenation responses during all three protocols compared with healthy subjects. Moreover, the patient with cognitive impairment showed CBF fluctuations that differed from those of the patient without cognitive impairment and the healthy subject. The cognitive impairment in CSVD patients is attributed to the decoupling of the neurons from the cerebrovasculature, which subsequently affects the autoregulatory capacity. This is the first investigation of cerebral hemodynamics in CSVD patients with concomitant mental disorders, demonstrating the great potential of combining the fNIRS and DCS for comprehensive evaluation of the neurovascular decoupling and diagnosis of cerebrovascular or psychiatric diseases.

## Closing remarks

With eight articles covering neurological evaluations in both infants and adults as well as the diagnosis of patients with cognitive impairment, we hope this Research Topic will provide timely and abundant information for scientists and clinicians working in the field of brain function and psychiatry, particularly those with fNIRS applications. We hope that the emerging fNIRS technologies, when combined with cortical activation paradigms and data analysis approaches, will have promising potential for brain functional evaluation and the diagnosis of mental conditions.

